# Crystal structure of human cysteamine dioxygenase provides a structural rationale for its function as an oxygen sensor

**DOI:** 10.1016/j.jbc.2021.101176

**Published:** 2021-09-08

**Authors:** Yifan Wang, Inchul Shin, Jiasong Li, Aimin Liu

**Affiliations:** Department of Chemistry, The University of Texas at San Antonio, Texas, USA

**Keywords:** sulfur oxidation, thiol metabolism, oxygen sensing and thiol regulation, nonheme iron, oxygenase, protein structure–function relationships, ADO, 2-aminoethanethiol dioxygenase, CDO, cysteamine dioxygenase, ERF-VII, group VII ethylene response factor, MDO, 3-mercaptopropionate dioxygenase, MSDO, mercaptosuccinate dioxygenase, PCO, plant cysteine oxidase

## Abstract

Cysteamine dioxygenase (ADO) plays a vital role in regulating thiol metabolism and preserving oxygen homeostasis in humans by oxidizing the sulfur of cysteamine and *N*-terminal cysteine-containing proteins to their corresponding sulfinic acids using O_2_ as a cosubstrate. However, as the only thiol dioxygenase that processes both small-molecule and protein substrates, how ADO handles diverse substrates of disparate sizes to achieve various reactions is not understood. The knowledge gap is mainly due to the three-dimensional structure not being solved, as ADO cannot be directly compared with other known thiol dioxygenases. Herein, we report the first crystal structure of human ADO at a resolution of 1.78 Å with a nickel-bound metal center. Crystallization was achieved through both metal substitution and C18S/C239S double mutations. The metal center resides in a tunnel close to an entry site flanked by loops. While ADO appears to use extensive flexibility to handle substrates of different sizes, it also employs proline and proline pairs to maintain the core protein structure and to retain the residues critical for catalysis in place. This feature distinguishes ADO from thiol dioxygenases that only oxidize small-molecule substrates, possibly explaining its divergent substrate specificity. Our findings also elucidate the structural basis for ADO functioning as an oxygen sensor by modifying *N*-degron substrates to transduce responses to hypoxia. Thus, this work fills a gap in structure–function relationships of the thiol dioxygenase family and provides a platform for further mechanistic investigation and therapeutic intervention targeting impaired oxygen sensing.

Thiol dioxygenases constitute a family of nonheme iron enzymes containing a ferrous iron center coordinated by three histidine residues (1, 2). The chemistry promoted by this class of enzymes is the S-oxygenation of thiols to sulfinic acids using molecular oxygen. Each enzyme of this protein family specifically oxidizes its own primary substrate(s). To date, thiol dioxygenases are found to oxidize two distinct types of substrates, conventional thiol-containing small molecules and recently discovered *N*-terminal cysteine-containing proteins. Cysteine dioxygenase (CDO) (3, 4, 5, 6), 3-mercaptopropionate dioxygenase (MDO) (7, 8, 9), and mercaptosuccinate dioxygenase (MSDO) (10) solely process small-molecule substrates; they are key players in regulating biological sulfur utilization and thiol metabolism. In contrast, thiol dioxygenases such as cysteamine (2-aminoethanethiol) dioxygenase (ADO) and plant cysteine oxidase (PCO) oxidize protein substrates. These posttranslational modifications target the oxidized proteins for degradation as a means employed by organisms to sense and respond to oxygen levels to help preserve oxygen homeostasis (11, 12). Oxygenation of the *N*-terminal thiol of group VII ethylene response factors (ERF-VIIs) by PCOs in plants destabilizes those factors, repressing the expression of anaerobic genes in the presence of sufficient oxygen (11). Among all thiol dioxygenases, ADO stands out for its broader spectrum of substrates (Fig. 1). It is the only enzyme of this protein family that oxidizes both small-molecule and protein substrates. Reactions catalyzed by other thiol dioxygenases are summarized in Scheme S1. ADO is the sole gene product responsible for converting cysteamine to hypotaurine (13). Its presence constitutes an important step in the metabolic scheme of l-cysteine or coenzyme A to taurine and the thiol metabolism cycle in mammalian cells (14). ADO was initially thought of as a sibling enzyme of CDO but oxidizing cysteamine, which is the only reaction in the thiol dioxygenase family that involves a small-molecule substrate without a carboxylate moiety (2, 13). Recent progress on ADO has pinpointed another layer to the importance of this enzyme in animals, which mirrors the role of PCO in plants. ADO is shown to modify *N*-cysteine signaling molecules, such as regulators of G protein signaling and the angiogenic cytokine interleukin-32. These oxidations potentially act as a more rapid mechanism to respond to hypoxia than transcriptional approaches, which are primarily modulated by hypoxia-inducible factor prolyl hydroxylases (12). Hence, in addition to regulating thiol metabolism, ADO initiates a transduction cascade, signaling hypoxia and preserving oxygen homeostasis in humans. Dysregulation of either process would cause serious diseases, such as defects in oxidative stress, neurodegeneration, autoimmune, cardiovascular function, etc (15, 16, 17, 18, 19). Consequently, ADO may be considered an emerging target for therapeutic intervention.

**Figure 1:**
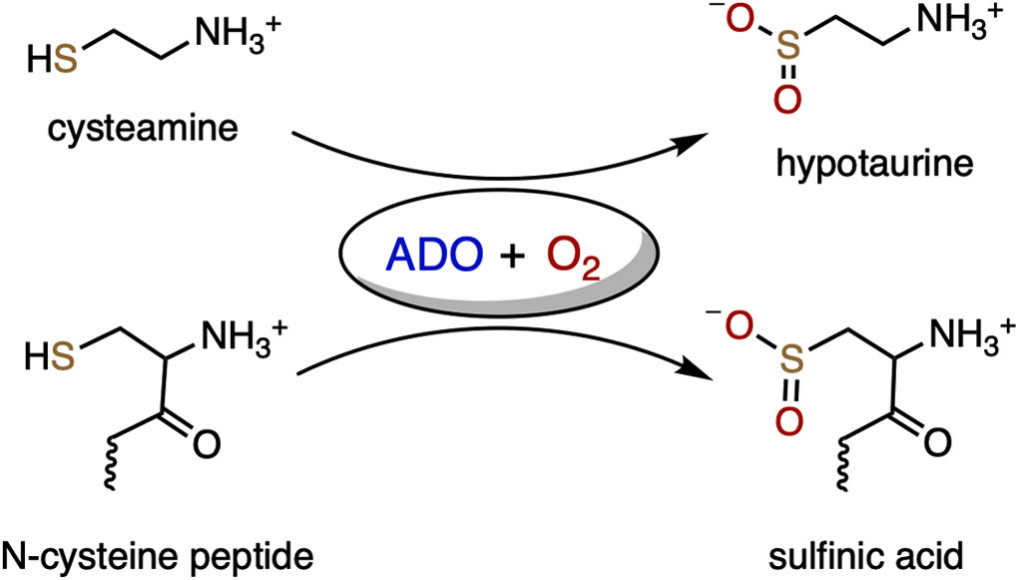
**Dual functions of ADO.** In the presence of oxygen, ADO oxidizes cysteamine to hypotaurine to regulate sulfur metabolism, as well as *N*-terminal cysteine-containing peptides to corresponding sulfinic acids to preserve oxygen homeostasis.

Compared with CDO, the only other human thiol dioxygenase, ADO, has not garnered much attention since its discovery more than half a century ago (20). Its significance, however, has been increasingly appreciated since the recent discovery that ADO functions as an oxygen sensor involved in *N*-degron pathways (12). In our previous studies, we cloned and expressed human ADO (hADO) and characterized its spectroscopic and kinetic properties (14, 21). We found that ADO can autocatalytically generate a Cys-Tyr cofactor (14). The Cys-Tyr cofactor, cross-linked between Cys93 and Tyr157 through a thioether bond, was first identified in CDO in 2006 (5, 6). The cofactor of ADO is formed by two residues close in sequence (Cys220 and Tyr222) and is predicted to be located on a different side of the iron center (14). For both enzymes, mutation of either Cys or Tyr could lead to a failure of cross-link formation and decreased catalytic efficiency by at least an order of magnitude (14, 22). The decreased activity, however, is not simply due to structural changes of the active site or the disruption of substrate binding but is associated with a critical role of the Cys-Tyr cofactor in catalysis. The unnatural amino acid incorporated variants, with the Tyr substituted by 3-chloro-tyrosine, 3,5-difluoro-tyrosine, and 3,5-dichloro-tyrosine through genetic code expansion, could generate halogenated, cross-linked cofactors (14, 23). The *K*_M_ values of these variants were comparable to wild-type enzyme's, and the active site was highly conserved with negligible modification. Still, the enzymatic activities were retarded. Therefore, the Cys-Tyr cross-linked cofactor is defined as a catalytic amplifier. The spectroscopic characterization of Fe(II)-ADO by EPR, Mössbauer, and absorption spectroscopies suggest that both cysteamine and a peptide substrate bind the ferrous center in the same monodentate fashion in contrast to the ES complexes of CDO and MDO. Additionally, ADO can readily form dinitrosyl iron complexes anaerobically in the presence of substrates (21). EPR and magnetic circular dichroism characterization of the Fe(III)-ADO by Brunold *et al*. (24) has led to a similar conclusion of the substrate-binding mode and a distinct secondary coordination sphere from CDO and MDO.

CDO and PCO are the most closely related members of the thiol dioxygenase family to ADO, and the crystal structures of both enzymes are available in various forms. The crystal structure of CDO was first reported from mice 15 years ago. It exhibits the β-barrel fold typical of the cupin superfamily with a Ni-substituted metal center and a cross-linked Cys-Tyr cofactor (5), which is identical in coordination to the Fe-containing form determined independently (6). Later, more CDO structures became available, such as structures of human, rat, and bacterial versions, proteins complexed with the substrate, gas molecules, as well as the structures with halogenated cofactors and without the cross-linked cofactors (6, 23, 25, 26, 27, 28, 29). The structures of PCO isoforms from *Arabidopsis thaliana* were reported very recently, including PCO2, PCO4, and PCO5, with a ligand-free Fe or Ni center (30, 31). PCOs share many common structural features with other members of the thiol dioxygenase family; however, their active site structures are distinct from CDO and MDO, presumably due to the need to bind protein substrates rather than small molecules (30). Yet whether these differences are indeed related and critical to the modification of protein substrates requires a direct structural comparison with ADO. ADO crystallization has proven to be challenging (32, 33). Thus, the three-dimensional structure of ADO is pivotal to advancing the thiol dioxygenase field to pursue a comprehensive structural investigation of how the active site accommodates two distinct types of substrates. hADO has been reported to exhibit multiple oligomeric states due to the formation of intramolecular disulfide bonds in air, which may contribute to the reluctance of hADO to be crystallized (14). We adopted the approaches of surface cysteine mutation and metal substitution to avoid the formation of heterogeneous oligomers. Herein, we report the first X-ray crystal structure of ADO.

## Results and discussion

### Crystallization of human ADO with surface cysteine modification

As described elsewhere, as-isolated ADO is composed of heterogeneous oligomer states presumably due to the presence of inter- and intramodular disulfide bonds (14, 32). While the significance of the disulfide bonds in ADO is worth future investigation, their presence yields inhomogeneous protein samples, which disfavor protein crystallization. Surface cysteine alteration through chemical modification or site-directed mutagenesis is often used to prevent the formation of disulfide bonds and facilitate protein crystallization (34, 35, 36, 37, 38). With the same crystallization condition described in the Experimental procedures, the Fe-containing wild-type hADO crystallized but did not diffract to a resolution sufficient for structural determination (32, 33). Since crystallization attempts with wild-type ADO did not lead to crystals diffracting to high resolution (32, 33), selective cysteine mutation was conducted in this work to generate ADO with homogeneous tertiary and quaternary structures for crystallization purposes.

**Figure 2:**
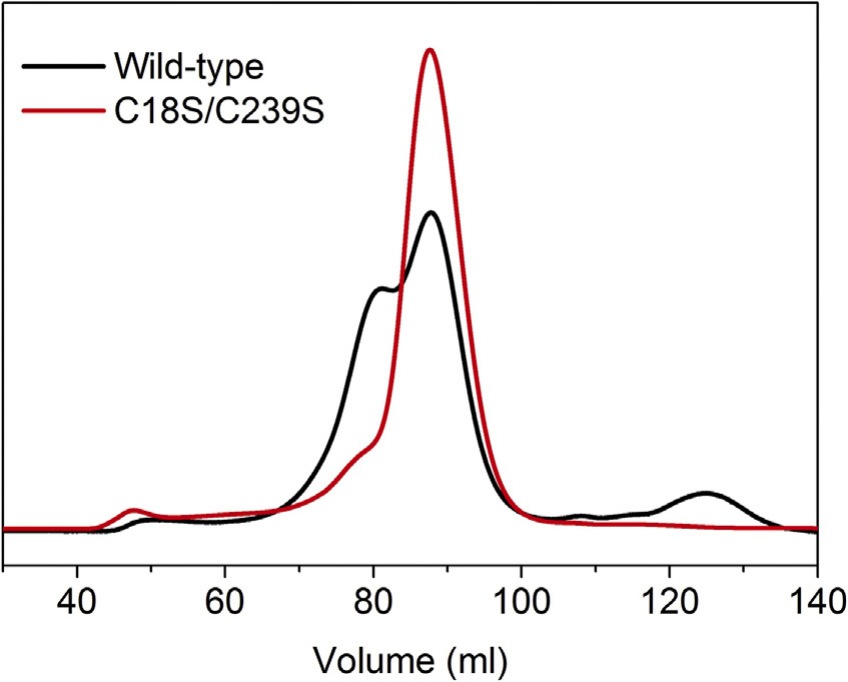
**Comparison of wild-type human ADO and the C18S/C239S variant by gel filtration chromatography.** The chromatogram was the result of gel filtration chromatography using a Superdex 200 column with 50 mM HEPES, 50 mM NaCl (pH 7.5). Black and red traces are for wild-type hADO and the C18S/C239S variant, respectively.

We identified Cys18 and Cys239 of hADO as surface residues that are not well-conserved among thiol dioxygenases. Only one out of six cysteine residues in hADO, Cys220 of the proposed Cys-Tyr cross-linked cofactor (14), is conserved in PCOs based on protein sequence alignment (Fig. S1). As shown by a structural model generated by the Phyre2 server (Fig. S2) (39), Cys18 and Cys239 are predicted to be located on the protein surface. These cysteine residues are most likely involved in forming intermolecular disulfide bonds. Hence, these two residues were mutated to serine. Indeed, the resulting double-site variant was more homogeneous than wild-type hADO. In turn, the monomer was the most predominant form in solution, as suggested by the significant peak in gel filtration chromatography (Fig. 2). In order to obtain hADO with a homogeneous metal center and prevent oxidation *via* the iron center, hADO cell culture was grown with minimal media supplemented with NiSO_4_ during induction of hADO expression. Hence, hADO was expressed as a Ni-bound, stable monomer, which was eventually crystallized, yielding high-quality X-ray diffraction for structural determination.

### The overall structure of ADO compared with other thiol dioxygenases suggests a high degree of structural flexibility

**Figure 3:**
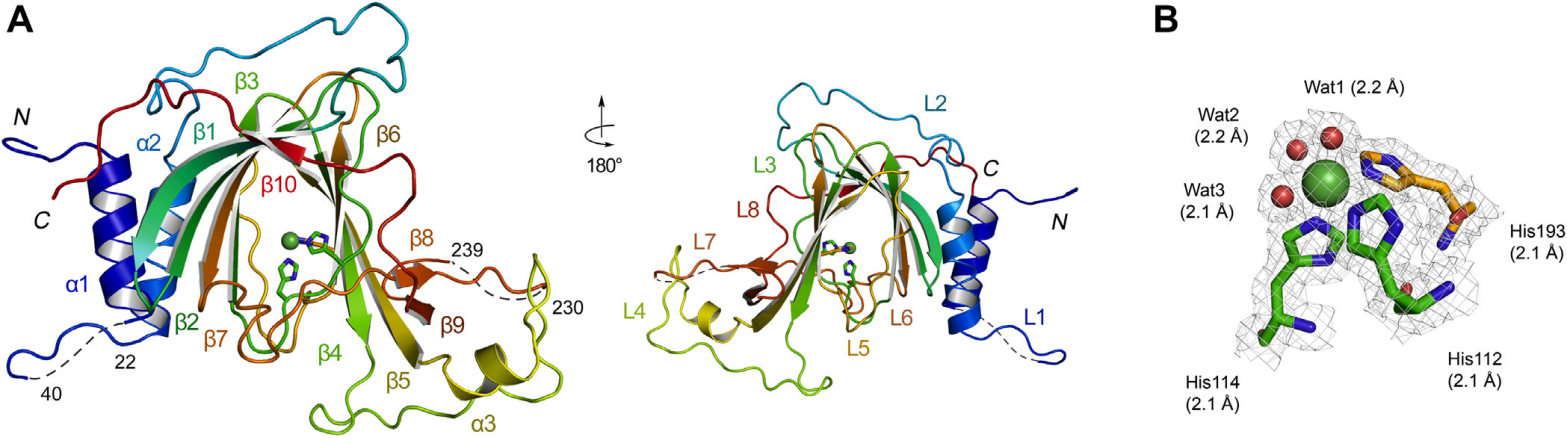
**Crystal structure of human ADO.***A*, the structure is composed of three α-helices, ten β-strands, and eight long connecting loops (L1–L8). Residues 22–40 and 230–239 are disordered. The *left* and *right panels* show the front and back ends of the β-barrel, respectively. The rainbow color begins with *blue* at the *N*-terminus to *red* at the *C*-terminus. Loop6 (β7-β8) and Loop8 (β9-β10) and extended β10 are proposed substrate recognizing regions. *B*, the nickel ion in this structure is hexacoordinated by a 3-His facial triad and three water molecules. The 2*F*_o_– *F*_c_ map is contoured at 1 σ, colored in *gray*. The metal–ligand distances (Å) are shown in *parentheses*.

**Figure 4:**
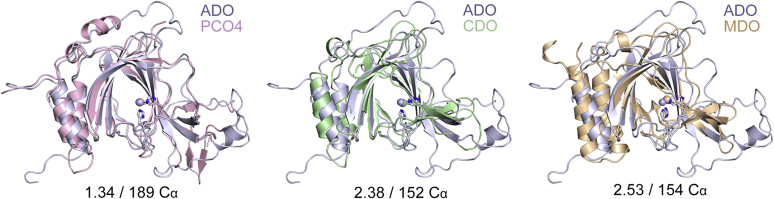
**Structural comparisons of ADO with other thiol dioxygenases**. Superposition of ADO (*purple*) with PCO4 (*pink*), CDO (*green*), and MDO (*wheat*) results in root mean square deviations (rmsd) of 1.34 Å 189 C_α_, 2.38 Å over 152 C_α_, and 2.53 Å over 154 C_α_, respectively. The PDB entries for ADO, PCO4, CDO, and MDO are 7REI, 6S7E, 6E87, and 4TLF, respectively.

The crystal structure of tagged hADO was determined at 1.78 Å resolution. The diffraction data were best interpreted in space group *C*222_1_, with *R*_work_ and *R*_free_ factors of 18.0% and 20.9%, respectively (Table 1). One monomer is present in each asymmetric unit. As shown in Figure 3*A*, most of the polypeptide residues are observed except the *N*-terminal tag, residues 22–40 and 230–239. The structure of hADO is composed of three α-helices, ten β-strands, and the remaining 62% of sequence are loops or disordered regions. Seven loops are over ten amino acids long, and three of which are more than 20 amino acids, including Loop 1 between α1 and α2, Loop 2 between α2 and β1, and Loop 4 between β4 and α3 (Table S1). In contrast, loop regions are generally short in proteins and 80% are shorter than ten amino acids (40). For loops with disordered (missing) residues or significantly higher *B*-factors than the overall peptide chain (>31.5 Å^2^), we define them as flexible loops. Loops 1, 2, 4, and 7 meet the criteria of flexible loops (Fig. 3*A*, right). These less common long loop regions indicate a significant degree of structural flexibility, as observed to a lesser degree in PCOs but not in thiol dioxygenases whose substrates are small molecules (Fig. 4). Hence, we interpret that the loop-rich structure accommodates the need for ADO to recognize and bind substrates of variable sizes, including proteins. Despite the flexibility, the core of the hADO structure is a “jelly roll” β-barrel that supports a catalytic center. The barrel is composed of two separate β-sheets, β10-β1-β2-β7-β3 and β5-β4-β6. The first β-sheet packs against α1 and α2, while the other β-sheet partially interacts with two short antiparallel strands, β8 and β9 (Fig. 3*A*, left). Enzymes of the cupin superfamily share low overall sequence similarity, while they all contain two highly conserved motifs and a less conserved intermotif region (1). Each of the three sections contributes two β-strands to construct a six-stranded β-barrel, as revealed in other thiol dioxygenases. The motifs and intermotif region are present in ADO, whereas they only furnish four β-strands (β3-β4-β5-β6). Loop 3 and Loop 5 are supposed to be β-strands in a cupin fold. Although Loops 3 and 5 are part of the well-ordered structures and not considered flexible loops, their presence reflects the increased overall flexibility of ADO relative to CDO and MDO. Hence, the overall structure of ADO is less rigid than other members of the superfamily.

**Table 1:** Data collection and refinement statistics

Process	Nickel-bound hADO C18S/C239S
Data collection	
Space group	*C*222_1_
Unit cell dimension	
a, b, c (Å)	54.9, 95.8, 117.6
α, β, γ (°)	90, 90, 90
Resolution (Å)	50.00–1.78 (1.81–1.78)^a^
Total no. of reflection	377,079
Unique no. of reflection	29,671
Redundancy	12.7 (10.2)
Completeness (%)	99.4 (97.9)
I/σI	27.3 (1.6)
*R*_merge_^b^ (%)	11.0 (100)
CC_1/2_	0.998 (0.790)
Refinement	
Resolution (Å)	47.62–1.78 (1.85–1.78)
No. of reflection	29,627 (2787)
*R*_work_^c^/*R*_free_^d^ (%)	18.0/20.9 (27.8/32.0)
No. atoms/*B*-factor (Å^2^)	
Protein^e^	1944/31.51
Nickel	1/19.35
Solvent	254/39.92
Root-mean-square deviations	
Bond lengths (Å)	0.007
Bond angle (°)	0.908
Ramachandran statistics	
Favored (%)	99.16
Allowed (%)	0.84
Outlier (%)	0.00
PDB entry	7REI

a Numbers in parentheses refer to data in the highest resolution shell.

b $R_{\text{merge}}=\sum \left|I_{\text{h}}-\left\langle I_{\text{h}}\right\rangle \right|\;/\sum I_{\text{h}}$, where *I*_h_ is the observed intensity and <*I*_h_> is the average intensity.

c $R_{\text{work}}=\sum \left|\left|F_{\text{obs}}|-\text{k}|F_{\text{cal}}\right|\right|\;/\sum \left|F_{\text{obs}}\right|$.

d *R*_free_ is the same as *R*_obs_ for a selected subset (10%) of the reflections that were not included in prior refinement calculations.

e Ordered residues: Arg3 – Arg22, Glu40 – Arg230, and Cys239Ser – Pro270.

In general, the overall structure of ADO is highly reminiscent of the PCO structures but with even more extensive flexible loop regions. A superimposition of hADO with PCO4 based on secondary structure matching indicates a root-mean-square deviation (rmsd) of 1.34 Å over 189 C_α_ (Fig. 4). The increased flexibility of ADO may arise from the requirement of ADO to handle more diverse protein substrates, while the PCOs are more specific to ERF-VIIs, with each PCO isoform having a particular substrate preference (41). In contrast, ADO is less similar to other thiol dioxygenases, with rmsd values of 2.38 Å over 152 C_α_ and 2.53 Å over 154 C_α_ for CDO and MDO, respectively (9, 27). This is anticipated since both ADO and PCO oxidize protein substrates, while the corresponding structural features are superfluous for other small-molecule thiol dioxygenases.

### A different active site architecture from small-molecule thiol dioxygenases

The structure of hADO shows a metal ion octahedrally coordinated by three histidine residues and three water molecules (Fig. 3*B*), which is a typical ligand scaffold for thiol dioxygenases. Since the recombinant protein was synthesized in M9 media supplemented with Ni^2+^, a nickel ion was modeled as the metal center. Although Fe is the native metal of thiol dioxygenases, Ni-substituted PCO and CDO were crystallized in the past and found to exhibit almost identical coordination as the Fe-bound proteins (5, 30). The 3-His coordination is a common feature and strictly conserved across the thiol dioxygenase superfamily (Fig. S1). The protein-derived ligands are His112 and His114 from Loop 3 and His193 from β6. These histidine ligands are located on one side of the β-barrel and do not directly interact with nearby residues. The metal center of ADO sits in a tunnel formed by the β-barrel. The front end (Fig. 3*A*, left) has a wider opening than the back end (right). Both ends are accessible to solvent. Presumably, the front end with Loops 6 and 8 binds the primary substrate, while the back end with Loop 5 is reserved for oxygen binding, as we recently found in a nonheme iron dioxygenase with a cupin structure (42).

**Figure 5:**
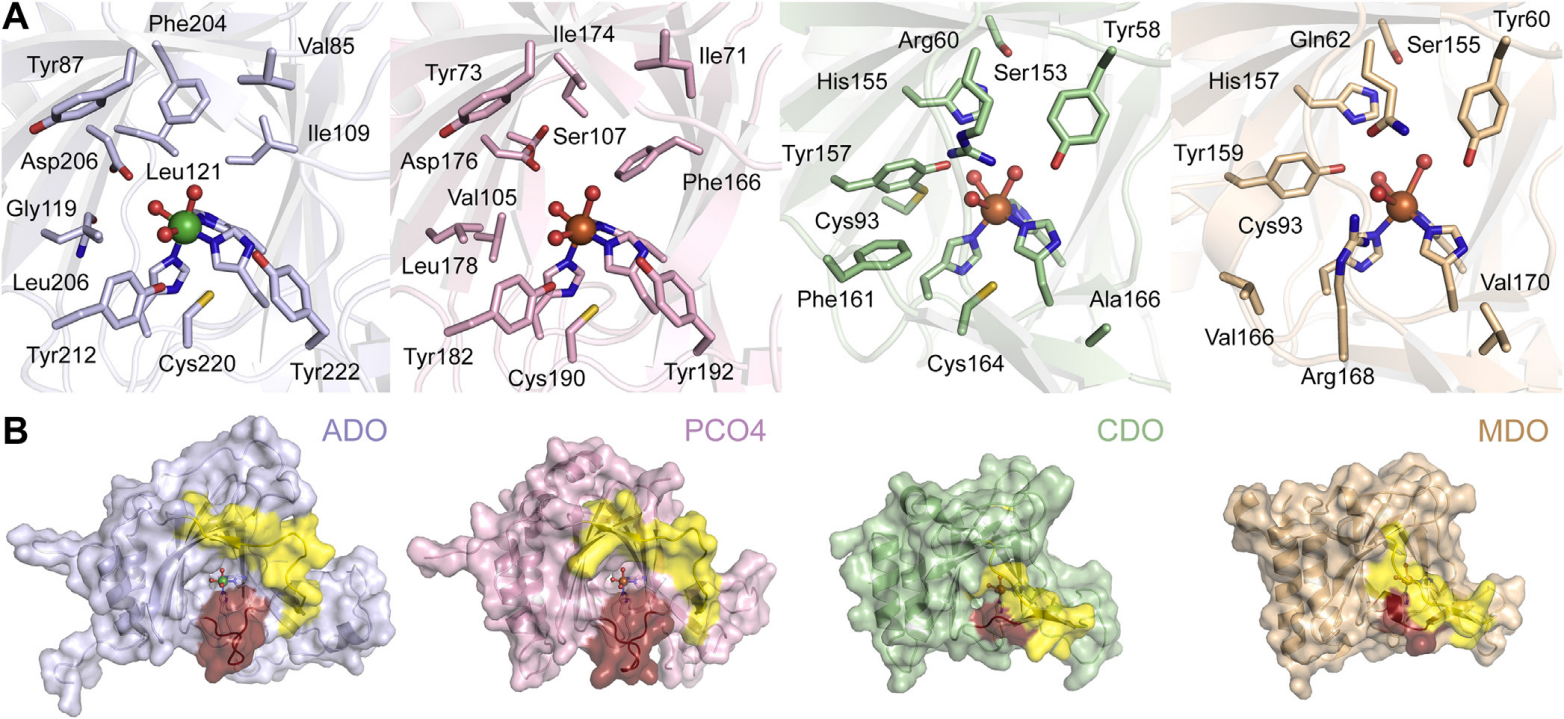
**Comparisons of active site architecture and cavity opening of human ADO with other thiol dioxygenases.***A*, active site architecture and *B,* surface representations of hADO, PCO4, hCDO, and MDO (from *left* to *right*). The active site residues of hADO are highly conserved in PCO4 but distinct from hCDO and MDO. In ADO and PCO, regions colored in *yellow* and *red* indicate the border region and the hairpin loop, respectively. However, in CDO and MDO, the corresponding *yellow* regions function as a “lid” of the catalytic cavity, and the corresponding hairpin loop regions are absent. Residues of the *yellow* regions are 249–261 in hADO, 220–231 in PCO4, 174–182 in hCDO, and 177–185 in MDO. Residues of *red* regions are 212–220 in hADO, 182–190 in PCO4, 161–164 in hCDO, and 163–168 in MDO. PDB entries 7REI, 6S7E, 6E87, and 4TLF (from *left* to *right*). The orientation is identical to the *left panel* of Figure 3*A*.

The active site architecture of ADO also highly resembles that of PCOs but shows more discrepancy when compared with other thiol dioxygenases that oxidize small molecules (Fig. 5*A*). In the active site of CDO and MDO, an outer-sphere Ser-His-Tyr (SHY) motif is identified and proposed to promote substrate binding and enhance catalysis. It may transfer protons for acid/base catalysis, although it is not catalytically essential (43, 44, 45). However, the SHY motif is missing in ADO and PCOs. Hydrophobic amino acids replace the Ser and Tyr, and an aspartate substitutes the His of the SHY in ADO and PCOs. Alteration of the aspartate in PCO4 (Asp176) led to low iron occupancy and decreased activities both *in vitro* and *in vivo* (30). In addition to its role in the SHY motif, the Tyr157 in CDO forms a cross-linked catalytic amplifier through a thioether bond with Cys93 when the protein needs to increase its catalytic power to boost metabolic processing of its redox-active substrate (25, 27). Such a cysteine is absent in ADO and PCOs. Gly119 in hADO and Val105 in PCO4 are found at the equivalent position. Hence, a cross-linked cofactor similar to that of CDO is not anticipated in ADO and PCOs but another cross-linked cofactor in ADO was found by a LC-MS/MS study (14) and is discussed below.

In addition to Tyr157 of hCDO, Tyr58 and Arg60 are key active site residues that stabilize substrate binding (25, 27, 46). The guanidinium moiety of Arg60 forms a strong salt bridge with the carboxylate of l-cysteine. Such a substrate-stabilizing arginine is functionally, but not structurally, conserved in MDO (*i.e.*, Arg168 of *Pseudomonas aeruginosa*), owing to the different positioning of its substrate carboxylate (9, 45, 47). Though the structure of MSDO has not been solved, it is predicted to also have a substrate-binding Arg as well (Arg66 of *Variovorax paradoxus* strain B4) (47, 48). Though essential for other small-molecule thiol dioxygenases, a substrate-binding arginine is not conserved in ADO (Tyr87 at an equivalent position), presumably because its small-molecule substrate (cysteamine) and protein substrates (*N*-terminal cysteine-containing peptides) do not have a free carboxylate group. Moreover, Tyr58 of hCDO is substituted by Val85 in hADO, which is analogous to Ile71 in PCO4. Overall, the similarity of the active site architecture of hADO with PCOs provides a structural rationale for its function as an oxygen sensor. Notably, a few active site residues are not conserved between ADO and PCO4: Gly119 *versus* Val105, Leu121 *versus* Ser107, Phe204 *versus* Ile174, and Ile109 *versus* Phe166. Although the differences occur on residues with chemically inert side chains, they could shape the active sites into different cavities for their respective protein substrates and may be relevant to the ability of ADO to process cysteamine.

In a ternary complex structure of hCDO, nitric oxide binds *trans* to a His ligand, which is equivalent to His112 of hADO. If the active site of ADO mimics that of CDO, water (Wat2) *trans* to His112 is expected to be replaced upon binding of the small-molecule substrate. However, the structure of ADO shows that the active sites of these two enzymes are significantly different, and Wat2 is in close contact with a negatively charged Asp206, which may suppress oxygen binding at this position. Additionally, a previous spectroscopic study suggests the iron center of ADO could simultaneously bind two nitric oxide molecules and cysteamine (21). Therefore, the structural data indicate that the oxygen-binding position in ADO may be distinct from that in CDO.

### An open catalytic cavity with loops designed for large substrates

A previously reported *At*PCO5 crystal structure shows an *N*-terminal His-tag from an adjacent protein molecule intruding into one end of the β-barrel (30). Such an interaction facilitated crystal packing and indicates that the end of the β-barrel is likely the active site entrance for *N*-terminal peptide substrates. Interestingly, such a phenomenon is not observed in ADO. Instead, residues 137–144 of Loop 4 intrude into the catalytic center of an adjacent, crystallographic symmetry-related molecule, forming interactions with residues at the active site entrance (Fig. S3*A*). Likewise, the intermolecular interaction observed in ADO could also assist crystal packing and potentially reflects the interaction between ADO and protein substrates. Additionally, extra electron density was found in the active site cavity, which was best fitted with a glycerol molecule from the cryoprotectant (Fig. S3*B*). The glycerol does not directly bind to iron but has interactions with iron-bound waters, providing another indication that the front end of the β-barrel is likely the substrate-binding cavity of ADO.

ADO has a more open cavity for substrate binding as compared with other thiol dioxygenases. The open cavity interacts with the intruding loop and a glycerol ligand. Figure 5*B* shows that the catalytic cavity is exposed to solvent in ADO and PCO4. In contrast, a *C*-terminal β-strand and a loop close the cavity entrance in CDO and MDO (highlighted in yellow), functioning as a “lid” to block the catalytic site. The corresponding region in ADO is Loop 8 and β10 (residues 249–261), which form a border of the β-barrel and leave the catalytic site accessible to ligands similar to what was observed in PCOs. This region is referred to as the border region hereafter. This structural difference can be rationalized by the need to handle distinct substrates. ADO and PCO must have an open and large cavity for large peptide-based substrates. In contrast, CDO and MDO only need to accommodate small molecules, and a relatively closed active site could protect the metal center from oxidation and adventitious binding of unwanted molecules. It is worth noting that even though the lid region of CDO and MDO shields one end of the β-barrel, it leaves a small opening behind the “lid” (Fig. S4).

Another structural feature conserved in ADO and PCOs but absent in other thiol dioxygenases is a hairpin loop at the catalytic entrance (Fig. 4*B*, colored in red, Loop 6 in hADO and residues 182–190 in PCO4). This hairpin loop is proposed to play a role in binding and recognizing protein substrates in PCOs (30); thus, it is not necessary for small-molecule-oxidizing enzymes. It shows sequence divergence among PCOs but is well-conserved among ADOs (Fig. S1), which is consistent with the finding that each PCO isoform has its respective biological role and is more selective and specific to certain ERF-VIIs (30, 41). In contrast, ADO is the only thiol dioxygenase identified so far to oxygenate *N*-degron substrates in mammals so that it may modify a broader scope of proteins with less substrate specificity. These protein-based substrates, however, may share certain structural features to be recognized by ADO. We speculate that the hairpin loop and the border region in ADO may function as two “belts” to fasten the protein substrates and enhance protein–protein interactions.

### A prospective Cys-Tyr cross-link in ADO

In our previous study of hADO, a Cys220-Tyr222 cross-linked cofactor was detected by high-resolution mass spectrometry after treating the iron-containing protein with excess cysteamine and O_2_ (14). Since the cross-link formation is between two adjacent residues, low-resolution techniques such as SDS-PAGE and gel filtration chromatography could not separate the cross-linked and uncross-linked forms. Similar to CDO, the cross-link is found to function as a catalytic amplifier, meaning the cofactor is not necessary for catalysis but boosts the catalytic efficiency when the thiol substrate rises to a high level and requires rapid depletion to resume proper thiol levels. Such a scenario frequently occurs in mammalian cells in the fed state, which does not appear in bacteria and plants. Although these two residues are strictly conserved in PCOs (Cys190 and Tyr192 in PCO4), we do not expect a cross-linked cofactor in PCOs because they neither metabolize small-molecule thiols nor experience similar fluctuations in the amount of redox-active thiol substrates that would need to be rapidly metabolized, like in mammalian cells.

The cross-link is not expected in this Ni-bound crystal structure, as its formation requires Fe, cysteamine, and O_2_. The distance between the sulfur of Cys220 and *ortho* carbon of Tyr222 is 4.0 Å in this cofactor-free structure, which is comparable to the distance in a structure of the uncross-linked form of rat CDO (3.8 Å, PDB entry: 6U4V) (28). The distances between the cysteine sulfur and tyrosine *ortho* carbon atoms are in a range of 3.7–4.2 Å in PCOs (Table S2). Free rotation of side chains could lead to a closer distance to form a prospective cross-link in an uncoupled oxidation event during cysteamine and O_2_ reactions. The distances of Fe-S (cysteine thiol) and Fe-O (tyrosine phenol) are 6.3 and 8.0 Å in hADO, similar to those of PCOs (Table S2). The distances of Fe-S and Fe-O are 4.2 and 5.0, as well as 4.4/6.0 and 3.9 in two reported uncross-linked CDO structures (6U4V and 6BPT) (23, 28). Cysteine and tyrosine are closer to the iron center in CDOs. However, unlike the cofactor of CDO, which sits on a β-strand of the cupin domain with much less mobility, Cys220 and Tyr222 of ADO are located at the end of the aforementioned hairpin loop (Loop 6). Since the hairpin loop is likely responsible for substrate binding and recognition (30), it is expected to be more dynamic, and upon substrate binding or during catalysis, it may move closer to the iron center, thereby allowing the formation of the cross-link. Upon forming a cross-link, the substrate selectivity of ADO may be altered, tuning the enzyme to prefer small-molecule substrates during the fed state in which cysteamine needs to be rapidly processed. Like the wild-type protein, the Fe-containing form of hADO with the double-site variant (C18S/C239S) did not generate diffractive crystals. Thus, the cofactor-containing form is anticipated to be crystallized in a distinct condition that is not yet known.

### Proline-rich regions potentially for substrate interaction

**Figure 6:**
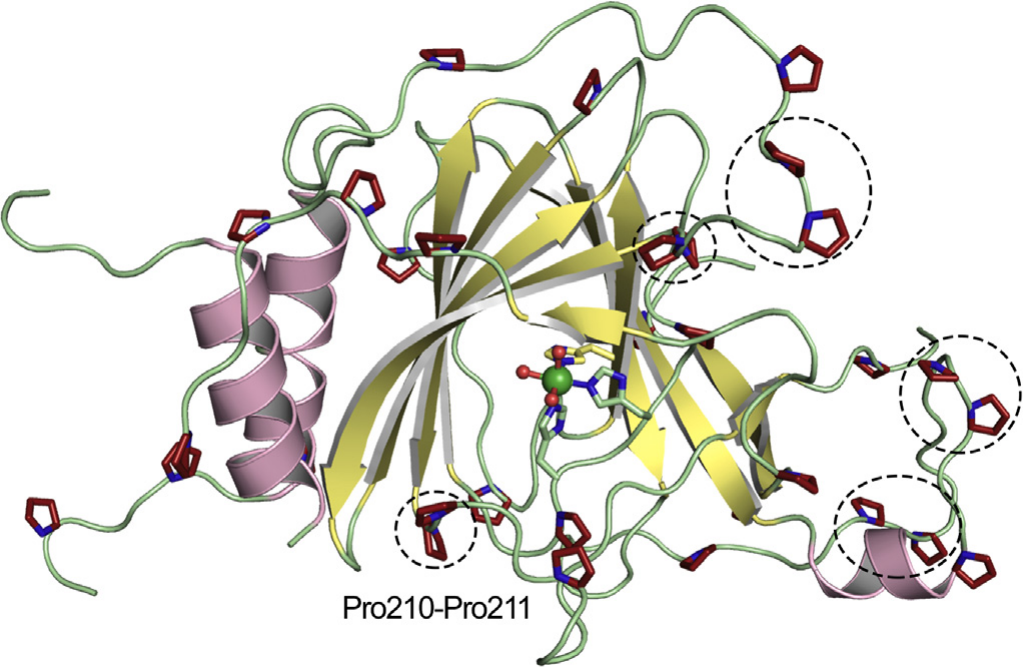
**Proline-rich regions in ADO.** All ordered proline residues are highlighted in *red*. Five di-proline motifs are highlighted in *dashed circles*. The secondary structural elements, helices, sheets, and loops are colored in *pink*, *yellow*, and *green*, respectively.

ADO is a proline-rich protein. There are 34 proline residues in hADO, accounting for 12.6% of the total amino acids. The prolines resolved in the structural data are highlighted in Figure 6. The number of prolines is less in PCOs (9.5% in PCO4) but more than double compared with other thiol dioxygenases such as CDO (4.5% in hCDO). Proline is known as a helix breaker. However, a proline–proline pair does not interrupt helices, as we have previously found in another nonheme Fe dioxygenase (42, 49). It is known that peptide bond synthesis is particularly slow with proline involved, and the production of two adjacent prolines often results in ribosome stalling (50, 51, 52). A reason should exist for incorporating a surfeit of prolines and di-proline motifs into ADO. The proline-rich domains have been found in some proteins that form interactions with their signaling partners (53, 54). Among the 34 prolines, 32 are clustered in loops and flexible regions, including the *C*-terminus, Loop 2, and the hairpin loop (Loop 6) (Fig. 6). Since proline has the least structural flexibility among the 20 genetically coded amino acids, the high number of proline residues would increase protein structural stability. Additionally, ten prolines are paired as “PP” di-proline motifs (circled in Fig. 6) in loop regions. Although only half of the prolines are conserved between ADO and PCO, the regions abundant in prolines align well between these two enzymes. A PP motif restricts the movement of its local structure and thus properly positions the side chains of catalytically essential residues. For example, a di-proline motif (Pro159-Pro160) is found after the Tyr157 in hCDO, which may assist a proper orientation of the phenol, thereby fulfilling its role in an outer-sphere motif and as a cross-linked cofactor. In the structure of ADO, Tyr212 follows a PP motif, Pro210-Pro211. The tyrosine and the di-proline motif are conserved in PCOs and are the starting point of the hairpin loop that is likely critical for substrate binding. Although the function of Tyr212 is unclear at this point, it is speculated to facilitate binding and catalysis of protein substrates considering its vicinity to the iron center and conservation between ADO and PCO. Hence, the PP motif could be designed to assist the correct positioning of Tyr212 or define the trajectory of the flexible hairpin loop. Therefore, the prolines appear to maintain the stability of the overall structure and specific critical residues' positions to contour the significant structural flexibility introduced by the extended loop regions.

## Concluding remarks

ADO is the only thiol dioxygenase that oxidizes two distinct types of substrates, *i.e.*, small-molecule thiols and *N*-terminal cysteine-containing proteins. The absence of a three-dimensional structure was a significant obstacle to an in-depth understanding of this enzyme. Hence, its crystal structure is of considerable interest for revealing structural factors that dictate functions. In this study, surface cysteine mutation and minimal media culture with a substituted metal resulted in the expression of a more homogeneous protein amenable to crystallization. The 1.78 Å resolution crystal structure described enabled direct comparisons with other thiol dioxygenases to reveal substrate specificity's structural rationale. Both the overall structure and the active site architecture of ADO are analogous to PCOs rather than human CDO. The open catalytic cavity and conserved loops gating the active site entrance are consistent with its function as an oxygen sensor that accommodates varying substrates. The Cys220-Tyr222 cross-link is not anticipated to be observed in this structure because of metal substitution. Aerobic processing of cysteamine by the iron-containing protein should be pursued before crystallization. An intriguing feature identified from the crystal structure is the proline-rich loop regions that potentially assist protein–protein interactions when dealing with a protein substrate and proper orientation of adjacent residues. Together, the first crystal structure of ADO adds new knowledge to the thiol dioxygenase family and provides a molecular foundation for mechanistic elucidation and therapeutic interventions.

## Experimental procedures

### Surface cysteine identification and mutagenesis

The predicted model of hADO was generated by Phyre2 (39) using PCOs for templates to locate potential surface cysteine residues of ADO. Cys18 and Cys239 were selected for mutagenesis to serine residues based on the predicted model. Primers were synthesized by Integrated DNA Technologies. The sequences of the forward primers are listed as follows with cysteine to serine codon underlined, and its reverse pairs are the reverse complement of the forward primers:

C18S: 5′– ATTGCACGTCAGGCAAGTCTGACCTTTCGTG–3′
C239S: 5′– GCAAGCAGCAGCGCAAGTGATCTGCCACGT–3′

The DNA sequence for codon-optimized human ADO is documented in supporting information. The gene was cloned to pET-28a plasmid with a cleavable His_6_-tag containing additional 30 amino acids at the *N*-terminus. PCR reactions were performed using the Phusion High-Fidelity DNA Polymerase kit purchased from Thermo Scientific. Successful generation of the double-site variant C18S/C239S was confirmed *via* DNA sequencing conducted by Eurofins Genomics.

### Protein expression and purification

His_6_-hADO was expressed in BL21 (DE3) (Merck), using standard M9 minimal media under kanamycin (50 μg/ml) selection. Cells were cultured at 37 °C in baffled flasks at 220 rpm until the OD_600_ reached 0.8 AU, at that time, the temperature was decreased to 28 °C prior to supplement with NiSO_2_ (20 μM) and isopropyl-β-d-thiogalactopyranoside (IPTG) (0.5 mM). Protein purification was derived from the reported procedures (14). Briefly, cells were harvested by centrifugation, disrupted, and clarified by centrifugation. hADO was isolated from clarified extracts using immobilized metal affinity chromatography with a prepacked HisTrap HP column (GE Healthcare). hADO was eluted from the column with 75 mM imidazole, desalted, and stored at –80 °C until use.

### Protein crystallization

Gel filtration with a Superdex 75 (26/65) column was used to further purify the protein in a buffer of 50 mM Tris-HCl and 50 mM NaCl at pH 7.6. The nickel-substituted hADO was concentrated to 67 mg/ml and mixed at 1:1 (v/v) with a crystallization buffer of 0.1 M BisTris-HCl (pH 5.5), 0.2 M Li_2_SO_4_, and 20% (w/v) PEG3350 using the hanging drop, vapor-diffusion method at 289 K. Microcrystals formed after one week and grew to an optimal size suitable for X-ray diffraction after 2 weeks. Crystals were cryoprotected with crystallization buffer containing an additional 20% (v/v) glycerol and then flash-cooled in liquid nitrogen.

### Data collection and structure determination

The hADO crystals were analyzed at the SSRL synchrotron beamline 9-2, and the diffraction data were collected at 100 K. HKL-3000 was used for data processing (55). A 1.78-Å resolution dataset was obtained at a wavelength of 0.97946 Å. Molecular replacement using PCO4 (PDB entry: 6S7E) as a search model was used to solve the phase with PHENIX.Phaser (56). Next, PHENIX.AutoBuild and PHENIX.Refine were used for model building and refinement (57). The data collection and refinement statistics are summarized in Table 1.

## Data availability

The data generated and analyzed in this study are included within the manuscript and supplementary data. The structural data have been deposited to the PDB databank with an entry code of 7REI.

## Supporting information

This article contains supporting information.

## Conflict of interest

The authors declare that they have no conflicts of interest with the contents of this article.
