# Supplementary material for: Crystal structure of human cysteamine dioxygenase provides a structural rationale for its function as an oxygen sensor
Source: J Biol Chem. 2021 Sep 8;297(4):101176. doi: 10.1016/j.jbc.2021.101176 (PMC8503633; doi:10.1016/j.jbc.2021.101176)
Supplement: Supplementary file 1 — Supporting Information [file mmc1.docx]

**Supporting Information**

**Crystal Structure of Human Cysteamine Dioxygenase Provides a Structural Rationale for its Function as an Oxygen Sensor**

<https://doi.org/10.1016/j.jbc.2021.101176>

Yifan (Amber) Wang, Inchul Shin, Jiasong Li, and Aimin Liu*

Department of Chemistry, The University of Texas at San Antonio, One UTSA Circle, Texas 78249, United States

The DNA sequence for the codon-optimized human ADO (hADO):

ATGGGCAGCAGCCATCATCATCACCATCATAGCAGCGGTCTGGTTCCGCGTGGTAGCGAAAATCTGTATTTTCAGGGTCATATGGCAAGTCCGCGTGATAATATGGCCAGCCTGATTCAGCGTATTGCACGTCAGGCATGTCTGACCTTTCGTGGTAGTGGTGGTGGTCGTGGTGCAAGCGATCGTGATGCAGCAAGCGGTCCGGAAGCACCGATGCAGCCTGGTTTTCCTGAAAATCTGAGCAAACTGAAAAGCCTGCTGACCCAGCTGCGTGCAGAAGATCTGAATATTGCACCGCGTAAAGCAACCCTGCAGCCGCTGCCTCCGAATCTGCCTCCGGTTACCTATATGCACATTTATGAAACCGATGGTTTTAGCCTGGGTGTGTTTCTGCTGAAAAGCGGCACCAGCATTCCGCTGCATGATCATCCGGGTATGCATGGTATGCTGAAAGTTCTGTATGGCACCGTTCGTATTAGCTGTATGGATAAACTGGATGCAGGCGGTGGTCAGCGTCCGCGTGCACTGCCTCCAGAACAGCAGTTTGAACCGCCTCTGCAGCCTCGTGAACGTGAAGCAGTTCGTCCGGGTGTTCTGCGTAGCCGTGCAGAATATACCGAAGCCAGCGGTCCGTGTATTCTGACACCGCATCGTGATAATCTGCATCAGATTGATGCCGTTGAAGGTCCGGCAGCATTTCTGGATATTCTGGCACCTCCGTATGATCCGGATGATGGTCGTGATTGTCATTATTATCGTGTTCTGGAACCGGTGCGTCCGAAAGAGGCAAGCAGCAGCGCATGTGATCTGCCACGTGAAGTTTGGCTGCTGGAAACACCGCAGGCAGATGATTTTTGGTGTGAAGGTGAACCGTATCCGGGTCCGAAAGTTTTTCCGTAA

**Table S1**. Long loops identified in hADO structure. Loop 1, 2, 4, and 7 have considerably higher average *B*-factor values than the overall peptide chain (> 31.5 Å^2^), and thus, are considered as flexible loop regions.

| **Loop** | **Number of residues/**  **number of ordered residues** | **Residue number**  **(position)** | **Average *B*-factor** (Å^2^)**/**  **number of atoms** |
| --- | --- | --- | --- |
| **1** | 25/8 | 22-46 (α1-α2) | 39.2/60 |
| **2** | 24/24 | 61-84 (α2-β1) | 42.3/191 |
| 3 | 14/14 | 104-117 (β2-β3) | 22.0/105 |
| **4** | 23/23 | 136-158 (β4-α3) | 36.5/176 |
| 5 | 15/15 | 177-191 (β5-β6) | 27.5/114 |
| 6 | 13/13 | 210-222 (β7-β8) | 29.1/108 |
| **7** | 17/9 | 226-242 (β8-β9) | 54.8/71 |
| 8 | 7/7 | 249-257 (β9-β10) | 25.8/74 |

**Table S2.** Distances related to prospective Cys-Tyr cofactors in uncrosslinked structures of thiol dioxygenases. d(S-C) refers to the distance between sulfur of cysteine and *ortho* carbon of tyrosine that potentially form a thioester bond; d(Fe-S) refers to the distance between the iron center and sulfur of cysteine; d(Fe-O) refers to the distance between the iron center and oxygen of tyrosine.

* The cysteine residue in this structure has two conformations, and thus, distances to both sulfur atoms are listed.

| **PDB entry** | **Protein** | **d(S-C) (Å)** | **d(Fe-S) (Å)** | **d(Fe-O) (Å)** |
| --- | --- | --- | --- | --- |
| 7REI | hADO | 4.0 | 6.3 | 8.0 |
| 6U4V | rCDO | 3.8 | 4.2 | 5.0 |
| 6BPT | hCDO | 3.2 / 3.7* | 4.4 / 6.0* | 3.9 |
| 6S7E | *At*PCO4 | 4.2 | 6.8 | 8.3 |
| 6S0P | *At*PCO4 | 3.7 | 7.1 | 8.1 |
| 6SBP | *At*PCO5 | 4.1 | 6.1 | 8.1 |
| 7CHJ | *At*PCO2 | 3.7 | 6.6 | 8.1 |
| 7CHI | *At*PCO4 | 3.9 | 6.3 | 8.2 |
| 7CXZ | *At*PCO5 | 4.0 | 7.1 | 8.7 |


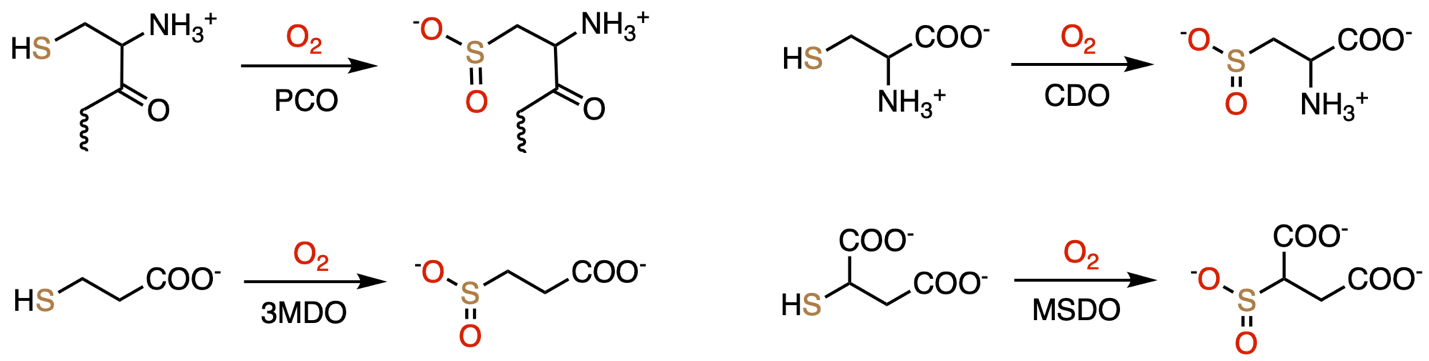


**Scheme S1.** Reactions catalyzed by thiol dioxygenases other than ADO.


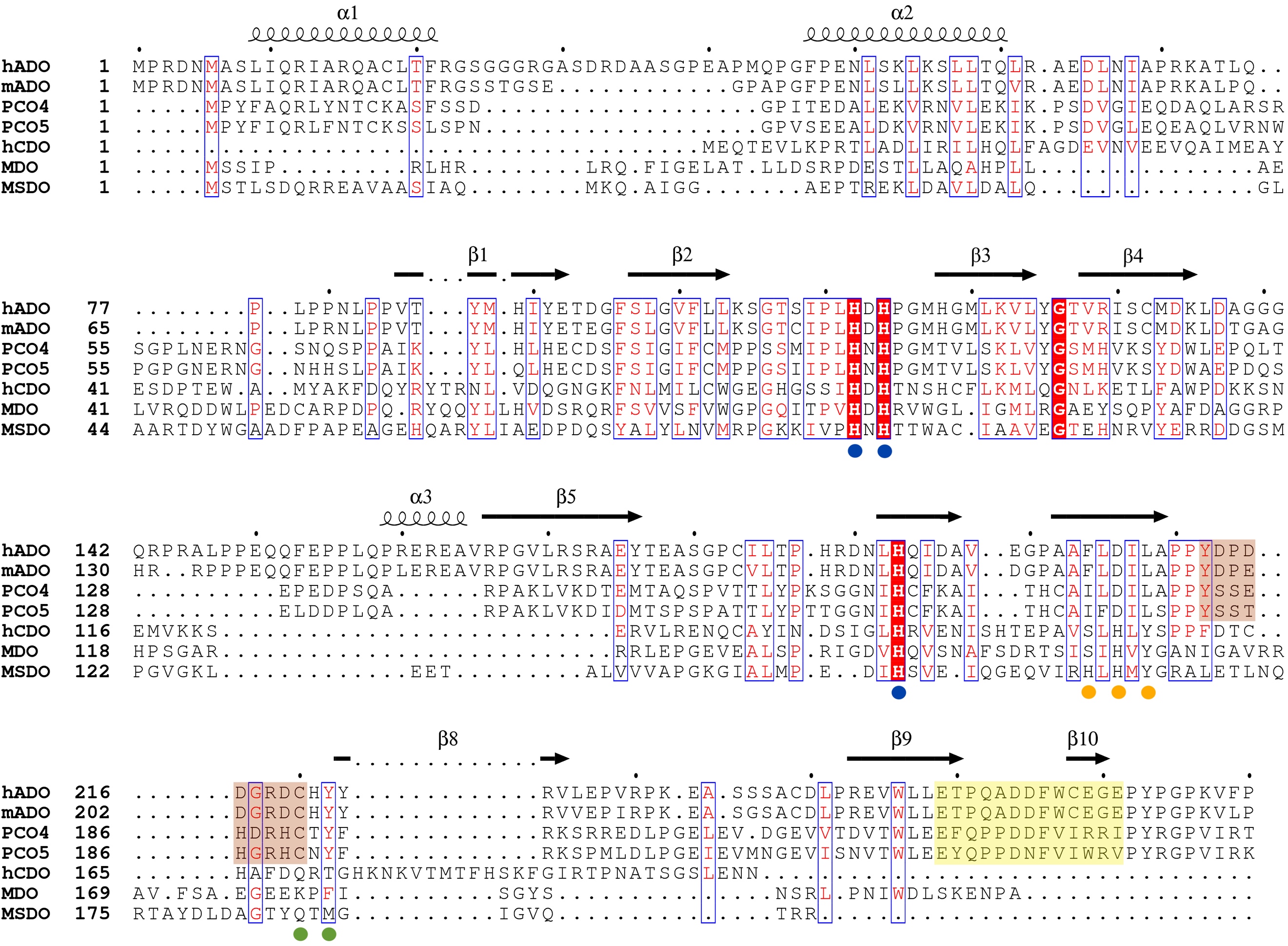


**Fig. S1.** Protein sequence alignment of ADOs and other thiol dioxygenases. The included sequences are ADO from *Homo sapiens* (NP_116193), ADO from *Mus musculus* (NP_001005419), PCO4 and PCO5 from *Arabidopsis thaliana* (NP_565980 and NP_191426), CDO from *Homo sapiens* (BAA12873), MDO from *Pseudomonas aeruginosa*, and MSDO from *Variovorax paradoxus* (WP_215247893). White characters with red background represent strictly conserved residues. Red characters represent residues of high similarity. Blue frames enclose similar residues across the sequences. The secondary structure elements are defined from the hADO structure. Conserved 3-His motif is marked by blue dots. Cysteine and tyrosine prospectively form crosslink in ADO are marked by green dots. Ser-His-Tyr outer-sphere motifs in CDO and MDO are marked by orange dots. A border region and a hairpin loop proposed for substrate binding and recognition in ADOs and PCOs are shaded in yellow and red, respectively.


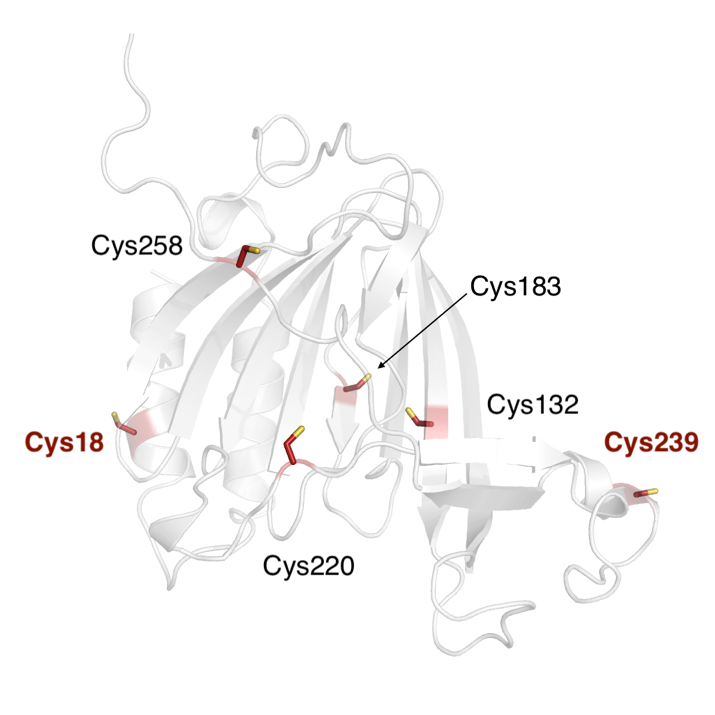


**Fig. S2.** Cysteine residues in the predicted hADO model generated by Phyre2 server. hADO contains six cysteine residues, as highlighted in red. Cys18 and Cys239 were selected for surface mutation.


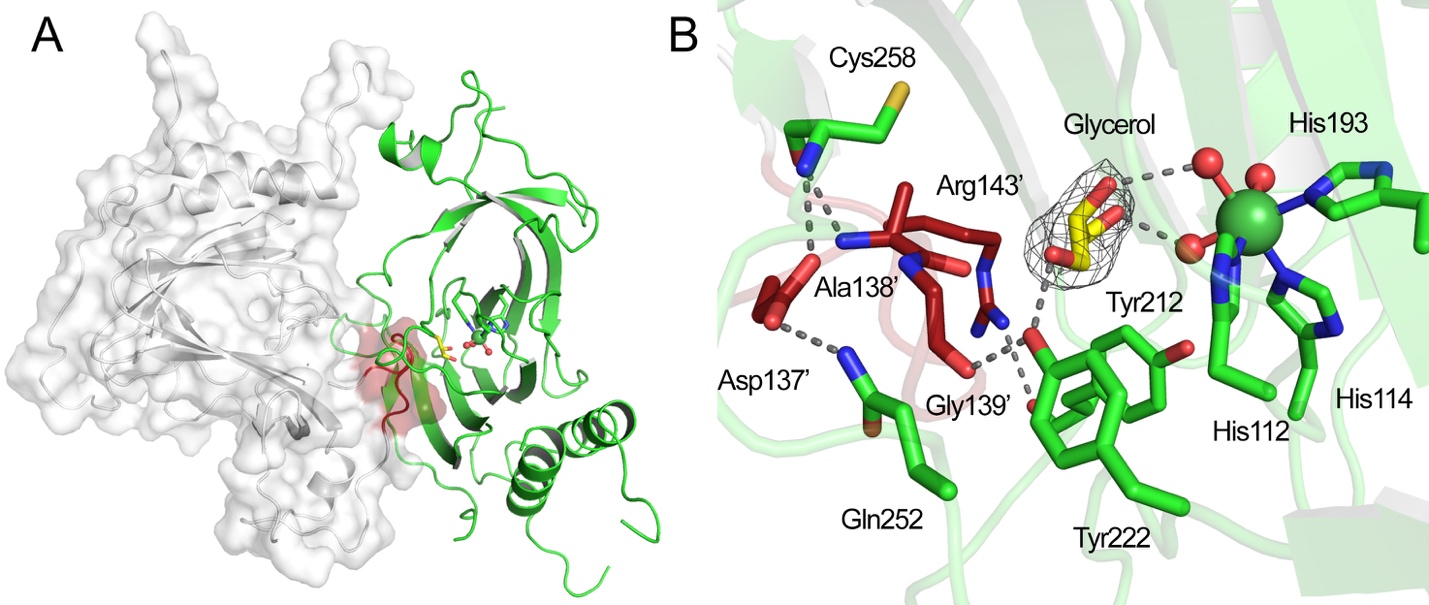


**Fig. S3.** Interactions between two crystallographic symmetry-related moclecules. (A). Part of Loop4 from one molecule (white surface representation) intrudes the catalytic center of an adjacent molecule (green ribbon diagram). The intruding part (colored in red) is composed of residues 137-144. (B) The active site cavity binds a glycerol molecule (shown in yellow carbons) which forms interactions with Tyr222 and iron-bond waters. Residues of the cavity entrance (green) form extensive interactions with the intruding residues (red) from the adjacent molecule. The 2*F*_o_– *F*_c_ map of glycerol is contoured at 1 σ, colored in gray.


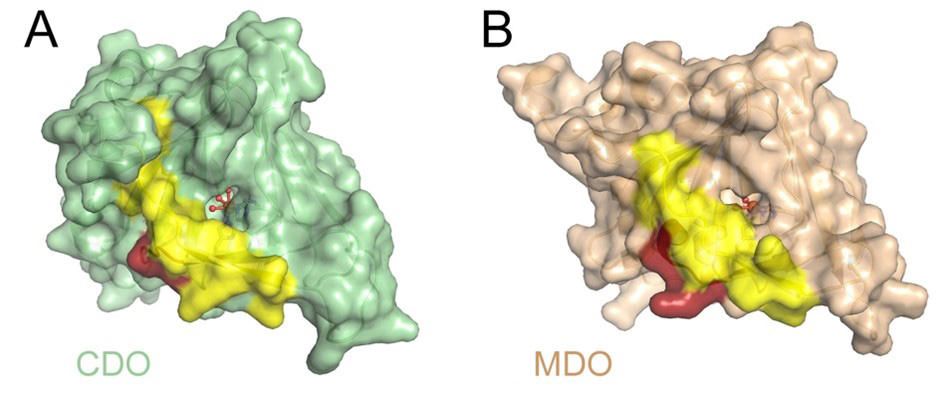


**Fig. S4**. A potential substrate entrance in CDO and MDO. (**A**) hCDO, PDB entry: 6E87; (**B**) MDO from *Pseudomonas aeruginosa*, PDB entry: 4TLF. The “lid” and the regions corresponding to a hairpin loop in ADO are colored in yellow and red, respectively.
